# Cost-Efficient Micro-Well Array-Based Colorimetric Antibiotic Susceptibility Testing (MacAST) for Bacteria from Culture or Community

**DOI:** 10.3390/bios13121028

**Published:** 2023-12-14

**Authors:** Huilin Zhang, Lei Wang, Zhiguo Zhang, Jianhan Lin, Feng Ju

**Affiliations:** 1 College of Environmental & Resource Sciences, Zhejiang University, Hangzhou 310027, China; 2 Key Laboratory of Coastal Environment and Resources Research of Zhejiang Province, School of Engineering, Westlake University, Hangzhou 310030, China; 3 Institute of Advanced Technology, Westlake Institute for Advanced Study, 18 Shilongshan Road, Hangzhou 310024, China; 4 Key Laboratory of Agricultural Information Acquisition Technology, Ministry of Agriculture and Rural Affairs, China Agricultural University, Beijing 100083, China; 5 Westlake Laboratory of Life Sciences and Biomedicine, School of Life Sciences, Westlake University, Hangzhou 310024, China

**Keywords:** antibiotic-susceptibility test, micro-well array chip, smartphone application, immunomagnetic separation, pathogenic bacteria

## Abstract

Rapid and cost-efficient antibiotic susceptibility testing (AST) is key to timely prescription-oriented diagnosis and precision treatment. However, current AST methods have limitations in throughput or cost effectiveness, and are impractical for microbial communities. Here, we developed a high-throughput micro-well array-based colorimetric AST (macAST) system equipped with a self-developed smartphone application that could efficiently test sixteen combinations of bacteria strains and antibiotics, achieving comparable AST results based on resazurin metabolism assay. For community samples, we integrated immunomagnetic separation into the macAST (imacAST) system to specifically enrich the target cells before testing, which shortened bacterial isolation time from days to only 45 min and achieved AST of the target bacteria with a low concentration (~10^3^ CFU/mL). This proof-of-concept study developed a high-throughput AST system with an at least ten-fold reduction in cost compared with a system equipped with a microscope or Raman spectrum. Based on colorimetric readout, the antimicrobial susceptibility of the bacteria from microbial communities can be delivered within 6 h, compared to days being required based on standard procedures, bypassing the need for precise instrumentation in therapy to combat bacterial antibiotic resistance in resource-limited settings.

## 1. Introduction

The invention of antibiotics in the 20th century has revolutionized modern medicine and launched their widespread use in curing clinical infections caused by a pathogenic bacterium or its community [1]. However, the overuse of antibiotics in the past few decades has accelerated the fast and uncontrollable development of clinically important antibiotic resistance in pathogenic bacteria closely associated with human health [2], such as extended-spectrum cephalosporin or carbapenem-resistant *Enterobacteriaceae* [3,4]. It is estimated that 10 million people will die of antimicrobial resistance by 2050 unless there is a prompt global response [5,6]. Therefore, cost-efficient antibiotic-susceptibility testing (AST) is of great importance for optimizing antibiotic usage in clinical treatment, guiding the rational selection of suitable antibiotics, and minimizing the emergence and spread of antibiotic resistance.

The conventional methods of AST (e.g., paper disk and broth dilution) are widely used in clinical diagnosis, and often time-consuming and labor-intensive due to tedious manual operations, such as strain isolation and bio-typing [7,8]. AST methods based on genotypic or phenotypic analysis [9,10,11] have been continuously developed to achieve the rapid test. Genotype-based AST methods can detect antibiotic resistance genes (ARGs) via a polymerase chain reaction or metagenomics sequencing [12,13,14,15]. However, only a few resistance genes are known to be firmly associated with phenotypic resistance, and newly acquired resistance mechanisms may not be detectable [9,16]. Phenotype-based AST methods, which evaluate the growth response of an organism or its specific viability to the presence of antibiotics, are most frequently used in real practices as the reference standard used by microbiology laboratories across the world [17,18]. Bacterial response to antibiotics can be typically evaluated based on physiochemical properties, such as pH [19], fluorescence [20], electricity [17,21], and surface-enhanced Raman scattering [22,23]. The methods mentioned, combined with well-equipped testing facilities, can achieve rapid AST of bacterial strains in 2 to 16 h. However, heavy instrumental investment and professional training are also required. Moreover, these rapid phenotype-based AST methods are usually low in throughput and not tailorable to emerging or alternative testing needs (e.g., multiple strains or antibiotics and tunable monitoring throughout) due to the special supporting facilities and immobilized instruments. Therefore, it is important to improve the inspection throughput of phenotype-based AST methods with portable instruments at an acceptable cost.

Microfluidic chips are increasingly popular AST tools that not only greatly improve the detection throughput but also sharply reduce the reagent cost of conventional AST with an adjustable volume from microliter to femtoliter [24,25,26,27]. In particular, droplet-based microfluidic AST methods can be operated with high throughput on the single-cell level [10,28,29]. Among them, a micro-well array is an excellent research tool for generating hundreds of independent wells without a precise process [30,31], allowing the tracking of bacterial growth in droplets over time in an antibiotic-amended medium [32]. However, in the application of AST, micro-well arrays are usually fabricated based on photolithography technology and accompanied by delicate instrumentation, such as a fluorescence microscope [30,33], resulting in difficulty in achieving the point-of-care in resource-limit conditions. It is ecologically attractive to develop a microarray chip using low-cost manufacturing technologies (e.g., 3D printing) compared with the photolithography technology and to integrate it with portable equipment, such as smartphone applications [34], for high-throughput testing to both meet convenience requirements and maximize its cost efficiency.

Although AST for pathogenic isolates or pure cultures is significant, it is imperative to acknowledge that, in practical, clinical, or environmental contexts, bacterial strains do not exist in isolation but instead in closely interconnected communities [35]. In addition, human pathogens require special isolation by laborious culturing, which is another key limiting factor for shortening the total turnaround time and cost-efficiency of AST [36]. In particular, clinically important and environmentally occurring pathogenic bacterial strains, such as *Salmonella* Typhimurium [37], need a special selective medium and complex process for isolation to gain the pure culture eventually. Immunomagnetic separation selectively enriches the target bacteria by directly separating them from the complex community, bypassing the requirement for isolation and facilitating further target cultivation [38,39,40]. The captured bacteria can also be more easily and more likely to be cultured from a community sample, as intense on-plate competition may weed out the less competitive but directly capturable species. The immunomagnetic separation with captured cells or their cultures provides a potentially efficient method to integrate into the AST process for routine screening of antibiotic resistant bacteria in different situations, such as environmental monitoring and healthcare facilities.

**Figure 1:**
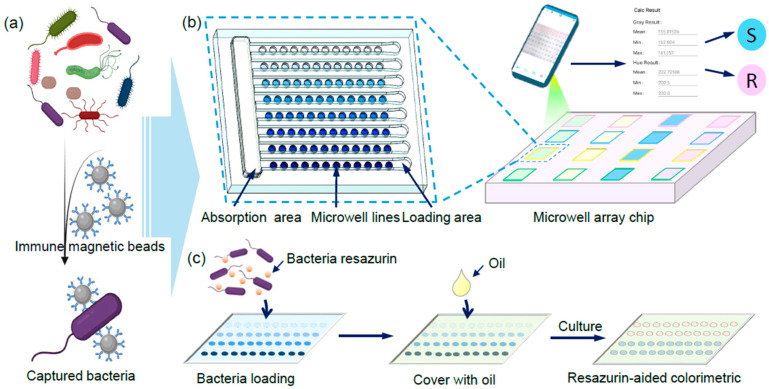
Conceptual sketch of an immunomagnetic separation-integrated micro-well array-based colorimetric antibiotic susceptibility testing (macAST) system with smartphone imaging. (**a**) Bacterial separation using immunomagnetic beads. (**b**) Resazurin-aided colorimetric AST based on a 3D-printed micro-well array chip and a smartphone APP. The micro-well array chip consists of sixteen sets of well arrays for simultaneously testing multiple bacterial strains and antibiotics. Eight micro-well lines with 12 micro-wells are also set in each well compatibly used for one kind of antibiotic with different concentrations. (**c**) The bacterial droplet generation process in the chip with different concentrations of antibiotics.

In this study, we reported a high throughput and robust micro-well array-based colorimetric AST (macAST) system and demonstrated its robust performance in practical tests against four bacterial strains with four representative antibiotics (Figure 1), generating consistent results with the classic broth microdilution methods. This system was further empowered by a self-developed offline application for smartphones, called Bioimage, for capturing and analyzing images to achieve rapid real-time AST and point-of-care in resource-limited conditions. More importantly, we broadened the applicability of the macAST to system from bacterial strains to complex environmental community by complementarily integrating immunomagnetic separation. The integrated system dramatically shortened the turnaround time for target isolation to 45 min and allowed for the direct capture of target cells from complex community samples, bypassing the need for cultivation (Figure 1).

## 2. Material and Methods

### 2.1. Preparation of Bacterial Strains

To verify the utility of the AST system, *Enterobacteriaceae,* listed as important within the critical group, was chosen as the test bacteria [41], two model strains, namely *E. coli* MG1655 and *S. typhimurium*, and two wastewater strains, i.e., *E. coli* EFE10 and *E. asburiae* CSS13, were chosen. The two wastewater strains were isolated from a local wastewater treatment plant and identified as extended-spectrum beta-lactamase (ESBL)-producing *E. coli* EFE10 (MALDI-TOF score: 2.3) and carbapenemase-producing *E. asburiae* CSS13 (MALDI-TOF score: 2.4) by Matrix-Assisted Laser Desorption Ionization Time of Flight Imaging Mass Spectrometry (MS-MS007, Bruker, Karlsruhe, Germany) [4]. All of the strains were revived by streaking on Luria–Bertani (LB) agar plates and stored at 4 °C. Their cultures were enriched in LB broth at 37 °C at 150 r/min for 16–24 h and then serially 10-fold diluted with Mueller Hinton (MH) broth to a specified concentration (~5 × 10^5^ CFU/mL) for antibiotic-susceptibility testing.

### 2.2. Design of Micro-Well Array Chip

The crucial component of the proposed macAST system was the micro-well array chip, which was fabricated using the well-established soft lithography technology based on 3D printing with rapid printing speed, simple operation, and low equipment cost [42,43]. The detailed procedure for fabricating the chip with polydimethylsiloxane (PDMS) has been described in our previous work [31]. In short, the pre-designed 3D model was printed using a commercial 3D printer (nanoArchR S140, Shenzhen, China) with high-precision (printing accuracy in the z-direction: 10–40 µm) based on Projection Micro Litho Stereo Exposure. Homogeneous PDMS and a curing agent from Dow Corning (Sylgard 184, Midland, MI, USA) were poured into the master model and then cured for future use. The micro-well array chip had a size of 56 mm × 56 mm × 3 mm and consisted of sixteen well arrays (Figure S1a,b). Each well array was composed of the loading area, the micro-well lines, and the absorbing area. In addition, the distance among well arrays was well-designed so that the sample could be loaded easily by hand using the conventional multichannel pipette. The depth of the eight micro-well lines was 0.20 mm, and there were 12 independent wells (R = 0.20 mm, H = 0.24 mm) evenly distributed on the bottom of the micro-well line for trapping the liquid to generate the independent droplet. The size of the micro-wells was designed specifically to balance the standard AST testing concentration and the minimal number of bacterial samples. In addition, the size of the micro-wells could be individualized in the design to capture more cell–cell heterogeneity with smaller volumes in the wells (Figure S1c). The micro-well array chip was first sterilized with 75% ethanol, followed by surface oxygen plasma (PCE-6, Shenzhen, China) treatment at 29.6 W for 30 s to gain the hydrophilic surface before use.

Antibiotics dilutions were prepared by diluting the stock antibiotics using water. There were 8 micro-well lines, 7 of the micro-well lines were filled with 0.5 µL of specific antibiotic dilution (64 mg/L, 32 mg/L, 16 mg/L, 8 mg/L, 4 mg/L, 2 mg/L, and 1 mg/L) and the last one was used as control without antibiotics. The micro-well array chip was then placed in a drying cabinet for 1 h at room temperature to ensure that the antibiotics were completely dry before preloading the antibiotics.

The number of bacteria in each of the wells was counted by injecting the *E.coli* MG1655 with a concentration of 5 × 10^5^ CFU/mL. The bacteria were first stained with SYTO@9 to label with the green fluorescent. After that, the number of bacteria in 60 wells was recorded using the motorized fluorescence microscope (Ni-E, Nikon, Tokyo, Japan; 20 × objective lens).

### 2.3. Antibiotic Susceptibility Testing in the Micro-Well Array Chip

For antibiotic susceptibility testing, MH broth was used as the standard AST growth medium. Bacterial suspensions were prepared to reach the standard AST bacterial concentration of ~5 × 10^5^ CFU/mL. The bacterial sample in MH broth medium was injected using a 10 μL conventional pipette. To load the sample, 0.5-μL of solution was dropped into the micro-well lines. After that, the mineral oil was dropped into the groove of the micro-well array to avoid the evaporation of the liquid. The antibiotic could be pre-loaded in each line in the micro-well array for further use. For the tests not using the pre-loaded antibiotics, bacterial suspension with resazurin was supplemented with the appropriate concentration of antibiotics before loading into the micro-well array chip. The mixed bacterial solution with different concentrations of antibiotics was prepared in the 0.2 mL centrifuge tubes. For loading the tested target, 0.5 μL of solution from the independent 0.2 mL centrifuge tubes was dropped into micro-well lines for further analysis. Similarly, all of the wells in the chip were covered by mineral oil to avoid evaporation of the culture medium. The micro-well array chip was then placed in the incubator at 37 °C and cultured for 5 h.

### 2.4. Cell Separation and Enrichment from Wastewater Samples

To extend the applicability of the 3D-printed AST chip beyond simple bacterial isolate samples, 1 L wastewater was sampled from a local wastewater treatment plant. The wastewater was first filtered with a 5 µm filter (Shengchao, Wenzhou, China) to prepare the sample for the future capture testing. One milliliter filtered wastewater samples were directly test for capture with 40 µg immunomagnetic beads. The spiked wastewater samples were prepared in three replicates by adding 1 mL of different concentrations of pure cultured bacterial cells into 9 mL of the wastewater supernatant and gaining a spiked sample with a bacterial concentration ranging from 3 × 10^3^ CFU/mL to 3 × 10^7^ CFU/mL.

To test the feasibility and efficiency of cell enrichment in complex wastewater samples, *S. typhimurium* was chosen as the target bacteria. The magnetic beads modified with monoclonal antibodies were used to capture the targets [44]. In brief, 40 µg immunomagnetic beads were added to 1 mL of pure cultured bacterial suspension or spiked wastewater to capture the target in a centrifuge tube. The centrifuge tubes were placed on the rotator to efficiently mix for 45 min. During the reaction, the immunomagnetic beads could combine with the target bacteria. The mixture was then separated by a magnetic separator, followed by washing five times using PBS solution. In addition, the capture efficiency (*E*) of the immunomagnetic beads was calculated by plating count using the following equation:

(1) $$E=\frac{N_{m}}{N_{m}+N_{s}}$$

where $N_{s}$ and $N_{m}$ were the numbers of bacterial cells attached to the immunomagnetic beads and in the supernatant, respectively [45].

### 2.5. Antibiotic Susceptibility Testing of the Captured Bacteria from Community

To achieve the direct AST of the target bacteria from the complex community, wastewater samples spiked with *S. typhimurium* with concentrations of ~5 × 10^3^ CFU/mL were prepared for further use. Forty-microgram immunomagnetic beads were added into 1 mL spiked samples and incubated for 45 min. After that, the tube was set into a magnetic separator (DynaMag-2, ThermoFisher, Waltham, MA, USA) for 2 min to discard the supernatant. Next, 0.5 mL of PBS solution was added into the tube to wash the captured bacteria and repeated five times. Then, 10 μL MH broth with 15 mg/L resazurin was used to resuspend the target. At last, the resuspended bacteria were added into the micro-well array chip and cultured for 5 h to report the AST result. In addition, to combine the immunomagnetic capture with AST, the captured *S. typhimurium* from pure culture with an initial concentration of ~5 × 10^5^ CFU/mL was tested the susceptibility to ampicillin, chloramphenicol, kanamycin, and tetracycline using MH broth in a 96-well plate.

## 3. Results and Discussion

### 3.1. Simulation of a 3D-Printed Micro-Well Array Chip with Trapped Droplets

**Figure 2:**
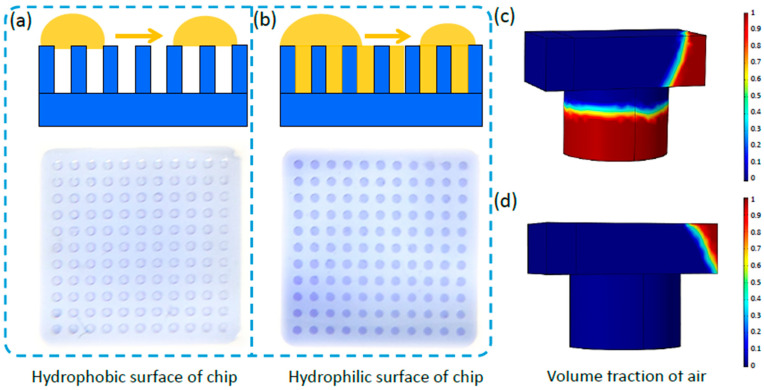
The simulation and analysis of the process of the water flow through the micro-well on a hydrophobic and hydrophilic surface in the micro-well array chip. (**a**,**b**) The state of water on the micro-array chip with a hydrophobic surface (**a**) and hydrophilic surface (**b**). (**c**,**d**) Simulation of the volume fraction of air during the process of water flow through the micro-well in the hydrophobic surface (**c**) and hydrophilic surface (**d**).

To make sense of the process of generating droplets in the PDMS-based micro-well array chip, a simulation was performed to optimize the conditions. The micro-well array developed in this study is an advantaged tool without a complex pump system to generate high-throughput independent droplets relying on the surface tension among the gas, liquid, and solid [46]. As a liquid with a large contact angle may not soak into a depression on a rough surface, and there may be a gas trapped between the liquid and solid as shown in Figure 2a. Therefore, the Cassie–Baxter model considering the mixed surface composite of gas and liquid was used, and the model equation was expressed as:

(2) $$cos\theta_{c}=f_{1}cos\theta_{1}+f_{2}cos\theta_{2}$$

where $f_{1}$ and $f_{2}$ represent the surface area fraction of the two mediums, and θ is the contact angle. In this study, the surface tension had a crucial effect on generating the droplets in the PDMS micro-well array. PDMS was hydrophobic material with a large surface contact angle (110°, Figure S3), making it easier for gas instead of water to fill the micro-well (Figure 2a). After the plasma treatment, the surface of PDMS become hydrophilic with a small surface contact angle (<10°), facilitating the liquid flow into and trap in the micro-well (Figure 2b). We also simulated and analyzed the process of the liquid flow through the micro-well on a hydrophobic and hydrophilic surface using COMSOL 6.0 based on the level set method. The simulation result showed that the gas phase volume fraction remains consistently above 0.5 when the fluid passes through the micro-well, when the surface contact angle of water and PDMS was set as 110° (hydrophobic state, Figure 2c), indicating that the micro-well could not be thoroughly filled with liquid. On the contrary, when the contact angle between water and PDMS was set as 10°, the gas phase volume fraction gradually approached zero as the liquid flowed into the micro-wells, indicating the liquid successfully filled the micro-wells (hydrophilic state, Figure 2d). Therefore, droplets could be generated in the hydrophilic micro-well array chip without precision control by a pump, and used for downstream high throughput AST. The material of the chip could be expanded, such as the photosensitive resin that could be directly fabricated by 3D printing. In this study, we chose PDMS for future testing.

### 3.2. Colorimetric Antibiotic Susceptibility Reporting through Smartphone Imaging of the Micro-Well Array Chip

**Figure 3:**
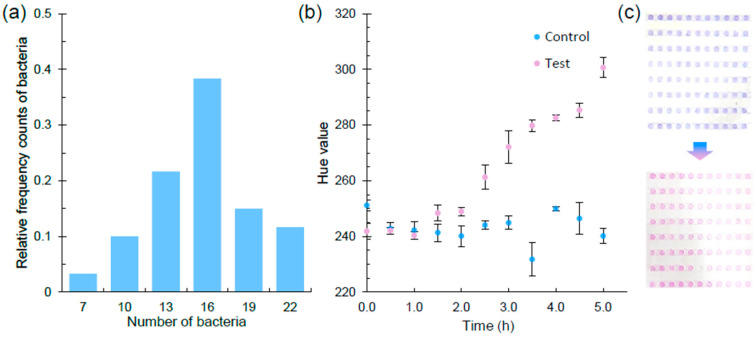
Initial number of bacterial cells and hue value change in the micro-well. (**a**) The bacteria distribution in the wells in micro-well array chip. (**b**) Hue value change when culturing the bacteria with resazurin in micro-well (data are represented as mean ± s.d., n = 20). (**c**) The color of wells changes from blue to pink in the micro-well array chip.

The AST results report for our proposed rapid AST system was based on the monitoring of the hue value in the micro-wells using Bioimage (v3.1), a smartphone APP that was developed in-house to automatically analyze the image data (https://github.com/emblab-westlake/Bioimage (accessed on 11 August 2022)). The cell number in the micro-wells (n = 60 wells) ranged from 7 to 22 (Average = 14.5 ± 3.7) in the micro-well array chip (Figure 3a). The change in color could be recorded by calculating the hue value based on Hue–Saturation–Lightness color space converted from the Red–Green–Blue model using the smartphone APP [47] (Figure 3b). Hue value classified colors within a wide range of [0, 2π], where 4/3π represents the pure blue and 2π represents the pure red, which will not be affected by different brands/models of mobile phones (Table S1). The color of a bacterial solution with resazurin would change from blue to pink with the normal growth of cells as the resazurin could be converted to resorufin (Figure 3c). During this process, the hue value would increase continuously with a large range (4/3π–2π) and is suitable to demonstrate the on-chip metabolic activity of examined bacterial cells. After culturing for 5 h, the hue value of the control group was around 240, while the positive group was up to ~300 and remained stable. Therefore, the threshold hue value was determined as 260 for further determination of the growth or inhibition for *E. coli* MG1655. We found that this time point and threshold hue value was also suitable for testing the other three bacteria, i.e., *E. coli* EFE10, *E. asburiae* CSS13, and *S. typhimurium*. In addition, a high-throughput phenotype-based AST method is usually performed optically in the micro-array wells [30,48,49]. The high-throughput micro-well arrays associated with colorimetric readout in AST also provided an appropriate point to combine with a smartphone, which has been developed as a simple-to-operate mobile health device [18], to achieve high-throughput phenotypic-based AST with cost-effective and portable detection apparatuses. Furthermore, the number of bacteria in the wells followed a Poisson distribution with λ = 15. In order to achieve an even hue value, ten micro-wells were selected for recording. Therefore, the number of wells in each line was set as 12 in the micro-well array chip to ensure that there were at least 10 micro-wells available for recording in case a few of the wells accidentally dried out.

Compared to the method used in the previous study [49], this micro-well array chip design was utilized with a minimal volume of bacterial samples (5 µL compared to the reported 200 µL) to enhance compatibility for future sample use. Additionally, a smartphone APP was developed to monitor bacterial growth by recording the hue value of the micro-wells, providing the facility for further integration with intelligent warning and monitoring system. The Bioimage APP can be used to record the change in hue value in the micro-wells by capturing an image of the micro-well array chip to determine the antibiotic susceptibility of the test bacteria. Resazurin can be converted to resorufin through a color change from blue to pink based on the active of aerobic bacteria [50]. This property makes it suitable for samples obtained from an aquatic environment where aerobic bacteria are common [51]. The color of droplets in wells often changes from blue to pink with the normal growth of the test bacteria in the PDMS micro-well array chip. Although the reported time of 5 h is not an inspiring advantage compared to other, much more rapid, methods based on Raman scattering (~2–3 h) [52], flow cytometry (~2–3 h) [53], and microscopy (2–4 h) [54], the results can be determined based on the colorimetric analysis, providing a convenient means for integrating with a smartphone and bypassing the need for precise instruments.

### 3.3. Micro-Well Array-Based Colorimetric Antibiotic Susceptibility Testing (MacAST) of Bacterial Strains

**Figure 4:**
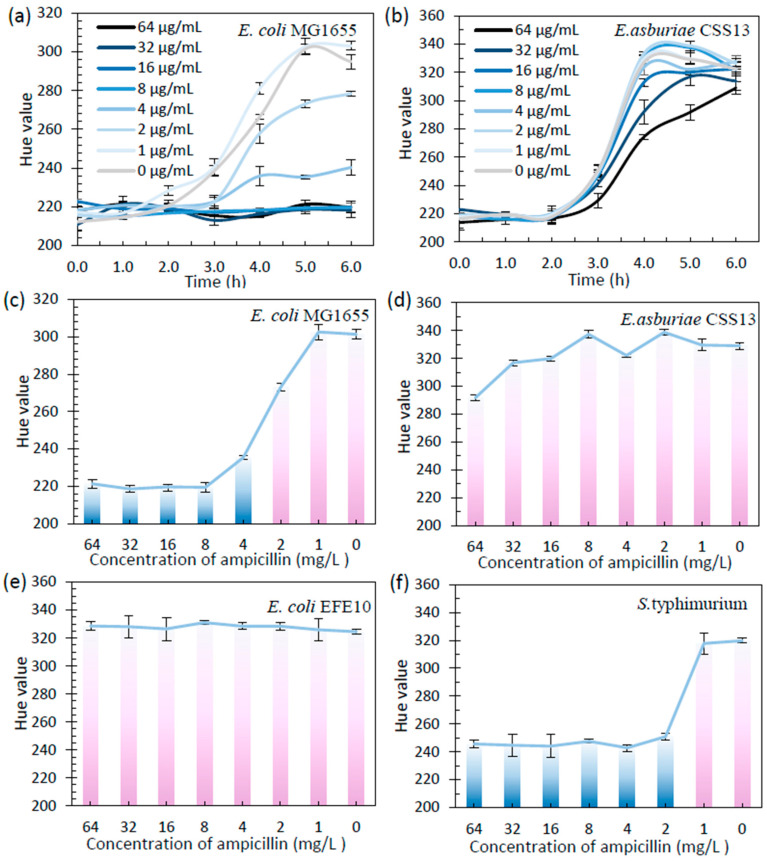
Micro-well array-based colorimetric antibiotic susceptibility testing (macAST) results of four bacteria in different concentrations of ampicillin. (**a**,**b**) Hue value records using Bioimage APP for *E. coli* MG1655 and *E. asburiae* CSS13 with the treatment of ampicillin (data are represented as mean ± s.d., n = 10). (**c**–**f**) AST result of *E. coli* MG1655, *E. asburiae* CSS13, *E. coli* EFE10 and *S. typhimurium* against ampicillin in 5 h; blue and pink bar represent the inhibited and normal growth of the bacteria, respectively (data are represented as mean ± s.d., n = 10).

The performance of our macAST system was validated on four *Enterobacteriaceae* strains including two model strains, namely *E. coli* MG1655 and *S. typhimurium,* and two wastewater isolates, i.e., *E. coli* EFE10 and *E. asburiae* CSS13. Metabolically active bacteria in the wells would convert blue resazurin to pink resorufin, which was used to determine the minimum inhibitory concentrations (MICs). The results were comparable to the MICs measured by the Clinical Laboratory Standards Institute (CLSI) guidelines. The Bioimage APP was used to continuously record the hue value during the culture of *E. coli* MG1655. There was a distinguishable difference in the hue value between the growth wells and the inhibited ones. The blue color of the well representing “Susceptible” (S), and the pink or colorless one representing “Resistant” (R). The MIC could be determined as the point with the most significant slope (Figure 4). After 5 h of culture, the wells in lines one to three had turned pink, with a hue value from 273 to 302, which indicated an obvious bacterial growth. In contrast, wells in lines four to eight, with no less than 8 mg/L of ampicillin, remained blue with hue value in the range of 218–235, representing the inhibition of bacterial growth. The other three antibiotics, chloramphenicol, tetracycline, and kanamycin, were also used for AST on micro-well array chip. *E. coli* MG1655 was susceptible to the four selected antibiotics, which was consistent with the conventional AST (Tables S2 and S3). *S. typhimurium* was also tested using the macAST system. The result showed that *S. typhimurium* was resistant to tetracycline, as the color of wells in lines one to seven became pink after 5 h of culture. In comparison, *S. typhimurium* was shown to be susceptible, with a determined MIC under ampicillin, chloramphenicol, and kanamycin treatment.

Next, we applied the above-demonstrated micro-well array chip to test the antibiotic susceptibility of clinically important extended-spectrum carbapenem or cephalosporin resistant *Enterobacteriaceae* strains of opportunistic pathogens isolated from raw wastewater. We observed that *E. asburiae* CSS13 growth was normal at concentrations of ampicillin from 0 to 64 mg/L, with hue values from 308 to 338, which showed that this strain was resistant to ampicillin (Figure 4), consistent with its experimental phenotype on the CHROMagar™ mSuperCARBA™ agar as a carbapenemase producer. After 5 h, This nosocomial pathogen was detected to be susceptible to the other three antibiotics, chloramphenicol, kanamycin, and tetracycline, with a MIC of 8, 2, and 2 mg/L, respectively. Likewise, we used the macAST system to promptly identify that the other wastewater isolate *E. coli* EFE10 showed multi-antibiotic resistance against ampicillin and tetracycline (Figure S4). This clinically important ESBL producer, however, was found to exhibit susceptibility to chloramphenicol and kanamycin treatment. In summary, the macAST achieved 16 out of 17 categorical agreements with the conventional AST results (Tables S2 and S3). The AST result for *E. asburiae* CSS13 with TET between the two methods (macAST vs. conventional AST) was inconsistent. This might be because *E. asburiae* CSS13 was not completely killed by TET at the concentration of 2 mg/L and 4 mg/L, and a few of the bacterial cells remained in the solution. In the macAST, during the culture for 5 h, the remaining cells might be in low activity, with the color of resazurin remaining blue. During the culture in the plate for 24 h, the remaining cells might recover growth and exhibited resistance to TET at the concentration of 2 mg/L and 4 mg/L.

### 3.4. Integration of Immunomagnetic Separation with macAST System (imacAST) for Direct Susceptibility Analysis in a Microbiota Sample

**Figure 5:**
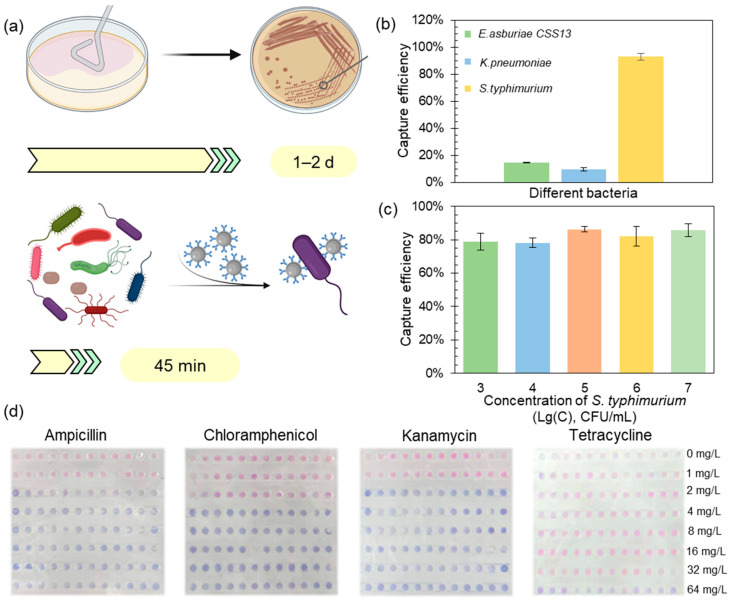
Immunomagnetic separation with macAST system used for complex microbiota. (**a**) Timeline comparing the current clinical workflow of the bacteria isolation to the immunomagnetic beads workflow. (**b**) Capture efficiency of the immunomagnetic beads for the target cells of *S. typhimurium* in contrast with the three other clinically important *Enterobacteriaceae* species (data are represented as mean ± s.d., n = 3). (**c**) Capture efficiency of the target bacteria from the spiked wastewater with different concentrations from 3 × 10^3^ CFU/mL to 3 × 10^7^ CFU/mL (data are represented as mean ± s.d., n = 3). (**d**) The images of AST result of the immunomagnetic beads captured *S. typhimurium* with an initial concentration of 10^3^ CFU/mL in 5 h.

Culture-independent enrichment methods based on immunomagnetic separation could dramatically decrease the turnaround time for isolating target bacterial strains from 1–2 d to only 45 min (Figure 5a). Here, we integrated the classic immunomagnetic separation method with macAST to upgrade the system to be compatibly applicable for the direct monitoring of target bacteria in complex microbiota samples. To achieve this goal, we first validated the competence of immunomagnetic beads to capture their designated targets, i.e., *S. typhimurium*, in the spiked wastewater. The bacterial capture capability of the immunomagnetic beads was determined using a transmission electron microscope (Figure S5). Meanwhile, two other bacterial strains, i.e., *E. asburiae* and *Klebsiella pneumoniae,* were comparatively tested as non-target bacteria to evaluate the specificity of the immunomagnetic beads. The capture efficiency of the beads reached 93% for the target *S. typhimurium*, while it was less than 20% for *E. asburiae* CSS13 *and K. pneumonia* (Figure 5b).

Further, three parallel experiments using *S. typhimurium* with different concentrations from 3 × 10^3^ CFU/mL to 3 × 10^7^ CFU/mL were spiked into the wastewater samples to simulate the scenario of a *S. typhimurium* outbreak. The recovery efficiency of the target cells ranged from 78% to 86% (Figure 5c). The result of the recovery efficiency was slightly lower than that for the pure cultures (93%), which might be due to the expected interference of the impurities in an environmental sample such as wastewater. Nonetheless, the relatively high recovery of the target bacteria demonstrated here enabled downstream testing of their antibiotic susceptibility at a relatively low biomass concentration. The AST results for the cultures and the immunomagnetic beads-captured *S. typhimurium* using the broth microdilution method in a 96-well plate were consistent, showing that normal AST could be conducted on the *S. typhimurium* captured by immunomagnetic beads (Figure S6). The captured *S. typhimurium* cells from the spiked wastewater samples with a concentration of 5 × 10^3^ CFU/mL were enriched for 100 times to conduct AST assays. The test result (ampicillin: S, chloramphenicol: S, kanamycin: S and tetracycline: R) after 5 h was consistent with that from the *S. typhimurium* suspension (Figure 5d) and the immunomagnetic beads-captured *S. typhimurium*, with an initial concentration of ~10^5^ CFU/mL, indicating that the AST assays could be conducted for samples with a low bacterial concentration (~10^3^ CFU/mL).

In summary, we demonstrated the feasibility to realize a direct analysis of target bacteria in a microbiota sample via the coupling of immunomagnetic separation with the Micro-well Array-based colorimetric Antibiotic Susceptibility Testing (imacAST) system. Compared with the other previously reported rapid AST methods, this system achieved rapid, convenient, high-throughput, and low-cost testing of low abundance pathogens in microbial communities (Table 1). Additionally, exploring approaches to reduce the time for culture isolation is tremendously important for shortening the total turnaround time of AST, which has not received enough attention in research. To adapt this proof-of-the-concept system for practical application, the following issues need to be considered. First, the capture efficiency can be improved by optimizing the performance of the antibody and parameters of the immunomagnetic separation [45], and the interference of the non-specific capture can be further reduced. Furthermore, considering that target bacteria usually exist within a community but not in isolation, the test results based on the potentially mixed sample can still meaningfully inform downstream antibiotic selection and treatment. Consistent with this argument, recent studies have demonstrated the direct filter capture of bacteria from urine samples [30,55], the filter-enriched bacteria constitute a mixed community sample that is recommended for antibiotic susceptibility testing, and showing the necessity of exploring effective approaches to optimize bacterial isolation processes. As a consequence, immunomagnetic separation could be integrated into the macAST system as a convenience and efficient method to combat the antibiotic resistant bacteria.

## 4. Conclusions

In this study, we advocated for and demonstrated the idea that bacterial isolation from a community should be considered for the future monitoring of antibiotic resistance. To move towards this conception, we proposed and reported an imacAST system that integrated immunomagnetic separation with high-throughput Micro-well Array-based Colorimetric Antibiotic Susceptibility Testing with a smartphone APP, reducing the total turnaround time from days to within 6 h and far outpacing the cost-efficiency of the AST that required culturing and precise equipment. In addition, immunomagnetic beads were used to capture and enrich the target bacteria in the real microbiota sample, providing direct AST of the target bacteria with low concentration from complex samples. In summary, the imacAST system is a cost-efficient platform with portable equipment for measuring bacterial susceptibility of sixteen combinations of bacteria strains and antibiotics from culture and communities with miniature reaction volume and high throughput, providing a promising alternative for fast and convenient AST, and promoting researchers to pay more attention to the methods for bacterial isolation and enrichment in AST.

We envision several ways to improve the utility of the imacAST system for future developments. Firstly, future validation of the system for capturing and susceptibility testing of target bacteria in clinical samples (e.g., urine) with low microbial diversity is warranted. Secondly, the capture efficiency can be improved by optimizing the performance of the antibody and parameters of the immunomagnetic separation to reduce the potential interference of the target bacteria in the AST results. Finally, the imacAST system could quickly separate the target bacteria from microbiota based on immunomagnetic separation. This can be coupled to the quantification of the target bacteria, as previously demonstrated [60,61], to determine the practical starting concentration of AST. In conclusion, we developed a cost-efficient imacATS system for bacteria from culture and community, and we foresee that the future improvement of the system will create a portable and tunable solution for conducting AST in research applications from environmental to clinical settings.

## Figures and Tables

**Table 1:** Comparison between our work and other previously reported rapid AST methods.

Fabrication of Chip	Time of Detection	Equipment	Throughput (per Chip)	Bacterial Isolation	Ref.
Paper-based	5 h	Multimeters	8	Classical culture (days)	[56]
Photolithography	2 h	Raman	1		[32]
Photolithography	5 h	Raman	64		[57]
Photolithography	2 h	Electrodes	1		[54]
Photolithography	2–3 h	Microscope	4		[33]
Photolithography	5 h	Naked eyes	12		[49]
Polypropylene	2–3 h	Smartphone	6		[58]
CNC-milled	~5 h	Microscope	4		[28]
3D printing	1.5 h	Spectrometer	6		[59]
3D printing	5 h	Microscope	6		[29]
—	2.5 h	Raman	—	Filtration (non-specificity)	[55]
Photolithography	4.5–5.5 h	Microscope	2		[30]
3D printing	5 h	Smartphone	128	Specific separation (<1 h)	This work

## Data Availability

Data are contained within the article and Supplementary Materials.

## Supplementary Materials

The following supporting information can be downloaded at: https://www.mdpi.com/article/10.3390/bios13121028/s1, Figure S1: The display of the micro-well array chip; Figure S2: The magnifying lens and the treatment of the images using Bioimage APP; Figure S3: Surface contact angle of the PDMS micro-well array chip; Figure S4: The images of AST result of the four bacterial strains in the micro-well array chip; Figure S5. TEM image of the immunomagnetic beads and bacteria captured by the immunomagnetic beads; Figure S6: The AST results for the cultured and the immunomagnetic beads-captured *S. typhimurium* using the broth microdilution method in a 96-well plate with different concentrations of antibiotics were consistent. Table S1. The comparison of the hue value of the same image captured by different brands/models of mobile phones. Table S2: MIC values (mg/L) and interpretive categories for *Enterobacteriaceae* species based on the CLSI guideline; Table S3: AST results of the test bacteria by the proposed system and 96-well plate.
