# Supplementary material for: Cost-Efficient Micro-Well Array-Based Colorimetric Antibiotic Susceptibility Testing (MacAST) for Bacteria from Culture or Community
Source: Biosensors (Basel). 2023 Dec 14;13(12):1028. doi: 10.3390/bios13121028 (PMC10741774; doi:10.3390/bios13121028)
Supplement: Supplementary file 1 [file biosensors-13-01028-s001.zip › biosensors-2727555-supplementary.pdf]

Supplementary Information

Cost-efficient micro-well array-based colorimetric antibiotic susceptibility testing for bacteria from culture or community

Huilin Zhang[1,2,3], Lei Wang[4], Zhiguo Zhang[2,3] Jianhan Lin[4], Feng Ju[2,3,5]*

[1] College of Environmental & Resource Sciences, Zhejiang University, Hangzhou, 310058, China

[2] Key Laboratory of Coastal Environment and Resources Research of Zhejiang Province, School of Engineering, Westlake University, Hangzhou 310030, China

[3] Institute of Advanced Technology, Westlake Institute for Advanced Study, 18 Shilongshan Road, Hangzhou 310024, China

[4] Key Laboratory of Agricultural Information Acquisition Technology, Ministry of Agriculture and Rural Affairs, China Agricultural University, Beijing 100083, China

[5] Westlake Laboratory of Life Sciences and Biomedicine, Hangzhou 310024, China

*** The corresponding author:**

Dr. Feng Ju (Assistant Professor)

No. 600 Dunyu Road, Sandun Town, Xihu District, Hangzhou, Zhejiang, PR China

E-mail: jufeng@westlake.edu.cn

Table of Contents

Experimental Section

Preparation of biochemical reagents and antibiotic stocks

The development of the smartphone APP (Bioimage)

Images capture and data analysis

Preparation of immunomagnetic beads

Conventional antibiotic susceptibility testing

Supplementary figure legends

Fig. S1. The display of the micro-well array chip

Fig. S2. The magnifying lens and the treatment of the images using Bioimage APP

Fig. S3. Surface contact angle of the PDMS micro-well array chip

Fig. S4. The images of AST result of the four bacterial strains in the micro-well array chip

Fig. S5. TEM image of the immunomagnetic beads and bacteria captured by immunomagnetic beads

Fig. S6. The AST result for the captured *S.* Typhimurium in a 96-well plate and the micro-well array chip

Supplementary Table legends

Table S1. The comparison of the hue value of the same image captured by different brands/models of mobile phones.

Table S2. MIC values (mg/L) and interpretive categories for *Enterobacteriaceae* species based on the CLSI guideline

Table S3. AST results of the test bacteria by the proposed system and 96-well plate

Experimental Section

Preparation of biochemical reagents and antibiotic stocks

Phosphate-buffered saline with 0.85% NaCl (PBS, 10 mM, pH 7.4; Sinopharm Chemical Reagent Co., Ltd, Shanghai, China) was used to prepare the reaction solutions. Resazurin (1%, w/w) was prepared in deionized water (R7017, Sigma, USA). Ampicillin obtained from Solarbio (Beijing, China) was prepared in a saturated $NaHCO_3$ solution, and chloramphenicol was prepared in ethanol. Tetracycline and kanamycin obtained from Macklin (Shanghai, China), was prepared in deionized water (18.2 MΩ·cm, Millipore, MA, USA). All these antibiotic solution (1%, *w*/*w*) were stored at -20°C after sterilization by filtration (0.2 µm). Luria–Bertani (LB) broth and Mueller–Hinton (MH) broth were purchased from Macklin (Shanghai, China). In addition, all chemicals were prepared and handled in a sterile environment to avoid contamination.

The development of the smartphone APP (Bioimage)

The Bioimage APP is designed to capture and calculate the hue values of target areas. The picture can be loaded for future analysis. The user can adjust the image to locate the perfect location in the picture using the function button for clipping, rotating, and zooming. After importing the image, the panel of this APP is shown to the users. The target area can be chosen manually or loaded from the saved process. Finally, both the hue value and the gray value can be recorded. In this study, we use the hue value for further analysis. The gray value can be used for analysis of the image using the fluorescence signal. The hue value is calculated based on the normalized RGB value

using the following equation:

$$max = MAX(R,G,B), min = MIN(R,G,B)$$

$$\mathrm{H} = \begin{cases} 0°, if\ max = min \\ 60° \times \frac{G-B}{max-min} + 0°, if\ max = R\ and\ G \geq B \\ 60° \times \frac{G-B}{max-min} + 360°, if\ max = R\ and\ G < B \\ 60° \times \frac{B-R}{max-min} + 120°, if\ max = G \\ 60° \times \frac{B-R}{max-min} + 240°, if\ max = B \end{cases}$$

Images capture and data analysis

The micro-well array chip was placed on a white surface to acquire a bright-field backdrop. The images could be captured by a smartphone equipped with 4X macro-lens (Hansi Optical Co., LTD., Zhejiang, China) under the lens of the smartphone. An application was developed in-house for determining the result of AST (https://github.com/emblab-westlake/Bioimage). The captured images were input into the smartphone APP named Bioimage (Fig. S2), which was specifically developed for recording the hue value. The hue value could be obtained using the insert algorithm based on the Hue–Saturation–Lightness (HSL) color space which was converted from the Red-Green-Blue (RGB) model after selecting the micro-well in the image.

Preparation of immunomagnetic beads

The synthesis of immunomagnetic beads has been well established in a previous study[1]. In brief, the anti-salmonella monoclonal antibodies (concentration: 1 mg/mL) from Abcam (Cambridge, MA, US) were first biotinylated through membrane dialysis using the long-arm biotin labeling kit. The magnetic beads with monoclonal antibodies, labeled with streptavidin conjugate, and immunomagnetic beads could be gained based

on the streptavidin–biotin binding.

Conventional antibiotic susceptibility testing

In this study, a gold-standard broth microdilution test was also performed to validate the results of on-chip AST developed by preparing four antibiotics with a range of concentrations in the bacterial suspension (128, 64, 32, 16, 8, 4, 2, 1, 0.5, 0.2, 0.1, and 0 mg/L). Generally, the initial concentration of bacterial cells was adjusted to ~5×10^5 CFU/mL, then, a 200 μL bacterial suspension was incubated in a 96-well plate in the presence of antibiotics at 37°C. The minimum inhibition concentration (MIC) could be determined based on the turbidity using a microplate reader (Varioskan LUX, Thermo Fisher, Waltham, MA, USA) or the naked eye after culturing for 18-24 h.

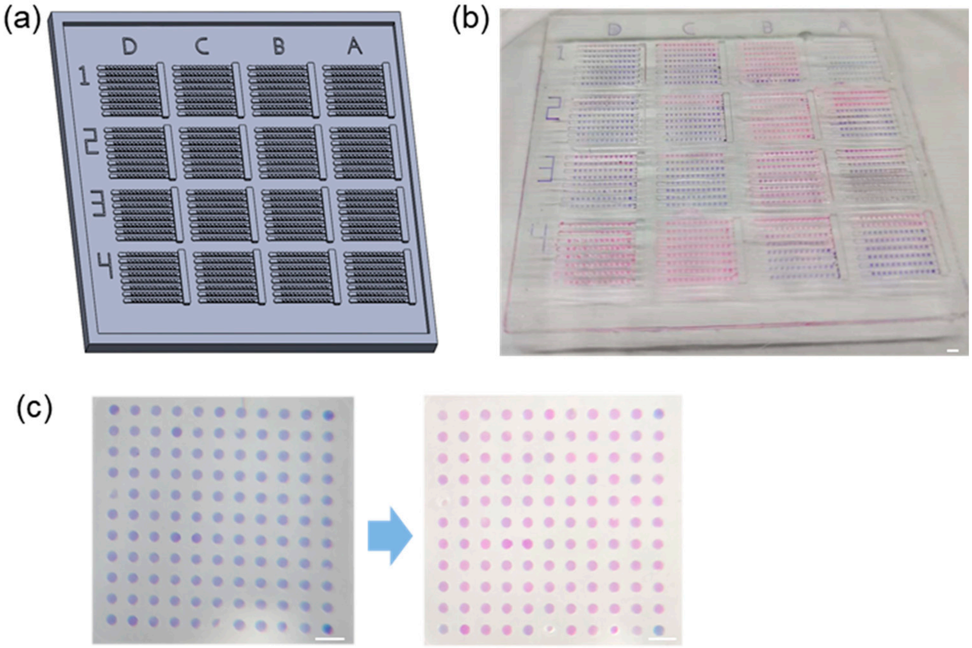

Figure S1. The display of the micro-well array chip. (a) The model for 3D printing; (b) the image of the micro-well array chip. (c) Micro-well plate with 121 wells for maximizing the bacterial dispersion (scale bar: 0.5 mm).

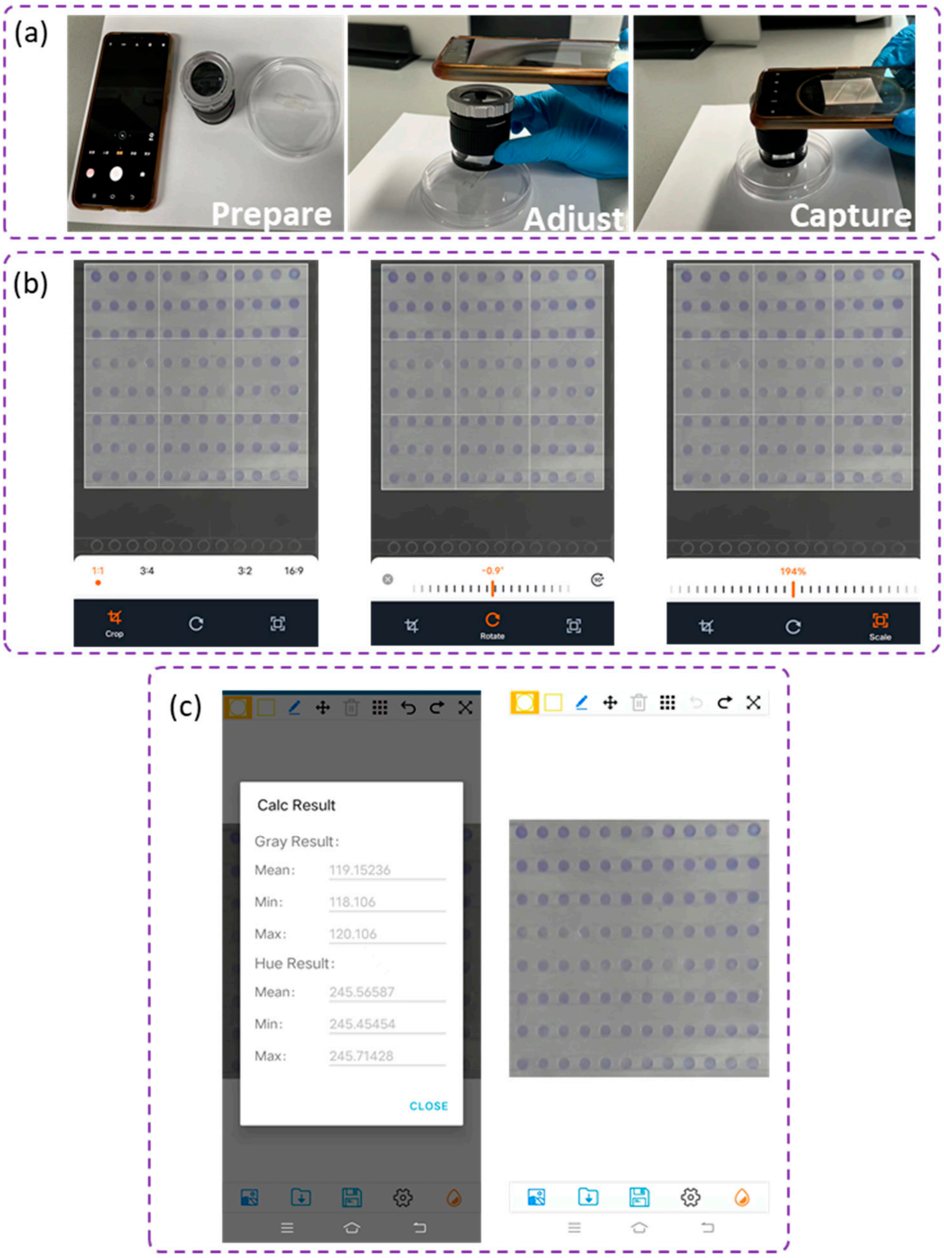

Figure S2. The magnifying lens and the treatment of the images using the Bioimage APP. (a) The capture process with the magnifying lens and smartphone. (b) Bioimage provides the users with the choice of adjusting the images using “crop”, “rotate”, and “scale” to gain a suitable angle or size before loading their images into the APP. (c)The panel of Bioimage. The target area can be chosen manually or automatically. The hue value and gray value can be reported simultaneously.

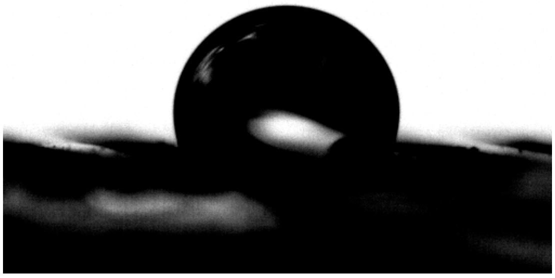

Figure S3. Surface contact angle (110°) of the PDMS micro-well array chip before plasma treatment determined using the contact angle measurement instrument (OCA 25, Dataphysics, Germany).

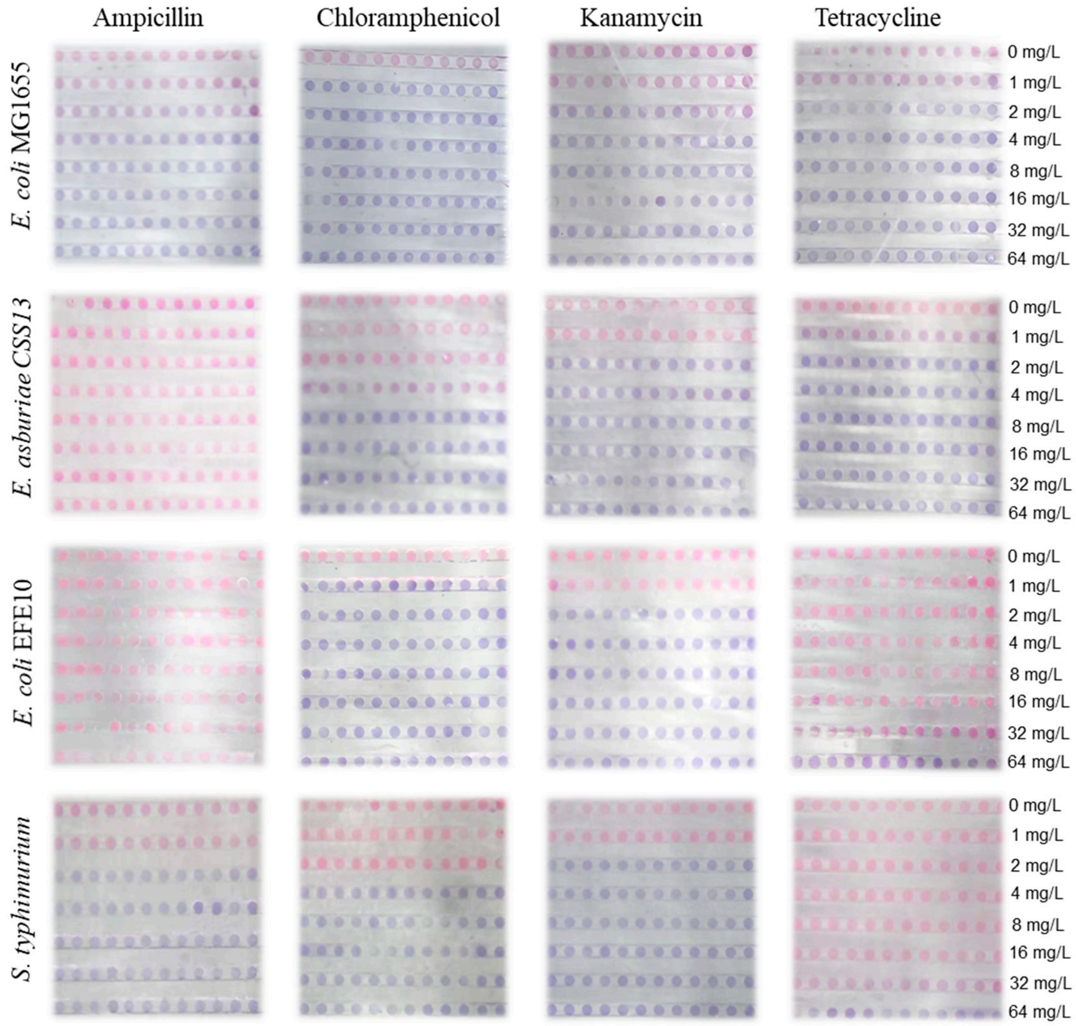

Figure S4. Images of AST results of the four *Enterobacteriaceae* strains against the four kinds of antibiotics in the micro-well array chip after culture for 5 h in the chip (micro-well in blue: the growth of bacteria was inhibited; micro-well in pink: the growth of bacteria was normal).

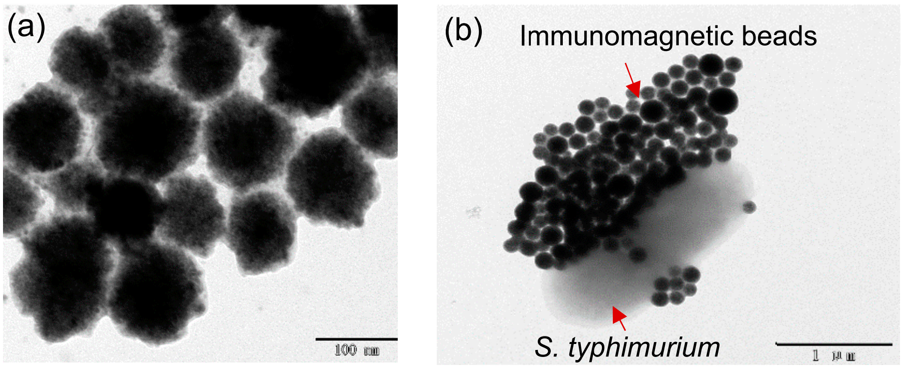

Figure S5. TEM image of the (a) immunomagnetic beads and (b) bacteria captured by the immunomagnetic beads.

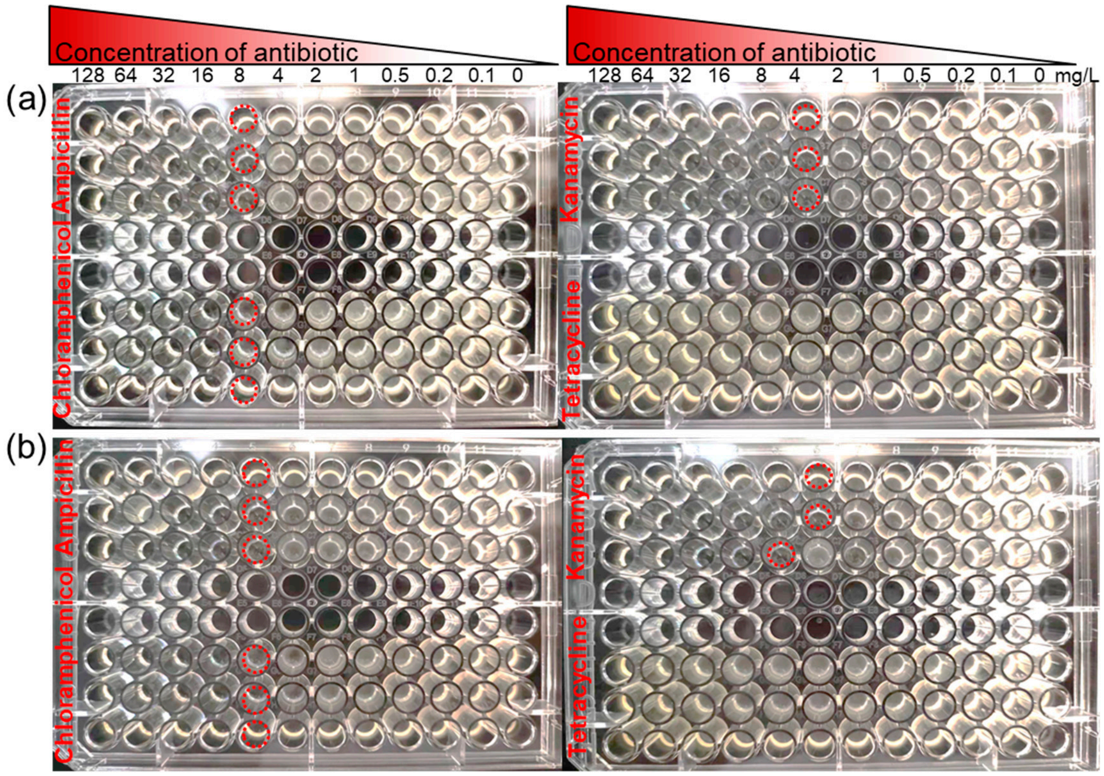

Figure S6. The AST results for cultured (a) and immunomagnetic beads-captured (b) *S.* Typhimurium using the broth microdilution method in a 96-well plate with different concentrations of antibiotics were consistent. The dotted line circles indicated the MIC value, which is the minimal concentration of the test antibiotic for inhibit bacterial growth.

Table S1. The comparison of the hue value of the same image captured by different brands/models of mobile phones.

**Mobile phones**	**Image**	**Size/Resolution**	**Hue value**
Apple iphone 13 mini	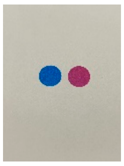	3024 × 4032/1.3M	211/329
Apple iphone 13 mini	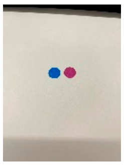	3024 × 4032/3.3M	211/329
vivo S7	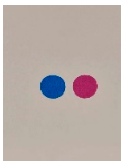	3456 × 4608/2.1M	211/329
vivo S7	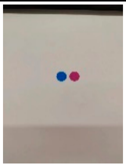	3456 × 4608/1.8M	211/329

Table S2. MIC values (mg/L) and interpretive categories for *Enterobacteriaceae* species based on the CLSI guideline.

**Antibiotics**	**Susceptible (S)**	**Intermediate (I)**	**Resistant (R)**
Ampicillin	<8	16	>32
Chloramphenicol	<8	16	>32
Kanamycin	<16	32	>64
Tetracycline	<4	8	>16

Table S3. AST results for the test bacteria from the proposed system and 96-well plate.

**Bacterial strain**	**Minimum Inhibitory Concentration (MIC) (mg/L) [Interpretation]**							
	**Microwell chip assay**				**Broth dilution in 96-well plate**			
	AMP	CHL	KAN	TET	AMP	CHL	KAN	TET
E. coli MG1655	4 [S]	<1 [S]	4 [S]	2 [S]	2 [S]	4 [S]	8 [S]	2 [S]
E. asburiae CSS13	>64 [R]	8 [S]	2 [S]	2 [S]	>128 [R]	8 [S]	2 [S]	8 [I]
E. coli EFE10	>64 [R]	1 [S]	2 [S]	>64 [R]	>128 [R]	4 [S]	8 [S]	128 [R]
S. typhimurium	2 [S]	4 [S]	2 [S]	64 [R]	8 [S]	8 [S]	4 [S]	>128 [R]

AMP: ampicillin, CHL: chloramphenicol, KAN: kanamycin, TET: tetracycline.

References

[1] F. Huang, L. Xue, H. Zhang, R. Guo, Y. Li, M. Liao, et al., An enzyme-free biosensor for sensitive detection of Salmonella using curcumin as signal reporter and click chemistry for signal amplification, Theranostics, 8(2018) 6263-73.
